# The Story of the Insane from Year to Year

**Published:** 1895-09-07

**Authors:** 


### THE STORY OF THE INSANE FROM YEAR TO YEAR.

Eighth Series.

### ALABAMA INSANE HOSPITAL AT TUSCALOOSA.

On the date of the last annual report this asylum contained 1,210 patients, 900 of these being white and 310 negroes. There were 334 admissions, 150 discharges, and 74 deaths. Dr. Searcy, when speaking of the recoveries, very truly says that those so discharged are usually sufficiently recovered to warrant their return and their trial at their homes. "A large proportion relapse. Of all persons declared insane, possibly not more than 10 per cent. recover sufficiently to be considered permanently cured." We fancy this is, perhaps, placing the recovery rate too low; but, unquestionably, patients are often discharged recovered who have not recovered, and this straining after the effect produced by the display of a high recovery rate is one of the reasons why insanity is not decreasing among us, and the next generation will suffer. Dr. Searcy thinks insanity is increasing in his State, as well as in civilised countries generally. His remarks on this subject are well put and very interesting, but space prevents our quoting them in full. The following sentences show one ground of his belief in the increase of insanity: "In civilised society humane sentiment makes us to continue and to propagate many lines and families, who in ruder, less civilised countries are promptly eliminated. In the present stage of our own more advanced civilisation we preserve and multiply the defectives whom we used to allow to go their natural eradication when we were savages." That is, we are in the period of the survival of the unfittest. Our morality is just sufficient to insist on our taking every possible care of our "defectives," as Dr. Searcy calls them; but it is not sufficiently developed yet to insist that it is positively criminal to bring these defectives into existence at all. We are told that much clinical work has been done, and abundant pathological material collected, and yet we find Dr. Searcy saying that the best remedial agent in the care of the insane is the diversion that comes of employment, and by far the best employment is that out of doors.

### IPSWICH BOROUGH ASYLUM.

At the close of the year there were 264 patients in residence, being 10 more than on January 1st. There were 95 admissions, 30 recoveries, 21 discharged relieved, and 22 deaths. The recoveries stand at 32·5 per cent. on the admissions, and the deaths at 8·4 on the average number resident. Cerebral and spinal diseases caused death in 8 instances, and consumption in 6. Hereditary influence caused the insanity in 23 of the year's admissions, and intemperance in drink in 4. The committee say "the state and condition of the asylum are satisfactory," and they make the same remark as regards the management. The average weekly cost per head is 9s. 4d.

